# Revealing links between gut microbiome and its fungal community in Type 2 Diabetes Mellitus among Emirati subjects: A pilot study

**DOI:** 10.1038/s41598-020-66598-2

**Published:** 2020-06-15

**Authors:** Mohammad Tahseen Al Bataineh, Nihar Ranjan Dash, Pierre Bel Lassen, Bayan Hassan Banimfreg, Aml Mohamed Nada, Eugeni Belda, Karine Clément

**Affiliations:** 1 College of Medicine, University of Sharjah, Sharjah, United Arab Emirates; 2 Research Institute for Medical & Health Sciences at University of Sharjah, Sharjah, United Arab Emirates; 3 Sorbonne University, INSERM, Nutrition and obesities: systemics approaches (NutrOmics), research Unit, Assistance Publique Hôpitaux de Paris, Nutrition department, Pitié-Salpêtrière hospital, Paris, France; 4 College of Engineering, American University of Sharjah, Sharjah, United Arab Emirates; 5 University Hospital Sharjah, Sharjah, United Arab Emirates; 6 Mansoura University, Mansoura, Egypt; 7 Integromics. Institute of Cardiometabolism and Nutrition (ICAN), Paris, France

**Keywords:** Microbial communities, Microbial genetics, Endocrine system and metabolic diseases

## Abstract

Type 2 diabetes mellitus (T2DM) drastically affects the population of Middle East countries with an ever-increasing number of overweight and obese individuals. The precise links between T2DM and gut microbiome composition remain elusive in these populations. Here, we performed 16 S rRNA and ITS2- gene based microbial profiling of 50 stool samples from Emirati adults with or without T2DM. The four major enterotypes initially described in westernized cohorts were retrieved in this Emirati population. T2DM and non-T2DM healthy controls had different microbiome compositions, with an enrichment in *Prevotella* enterotype in non-T2DM controls whereas T2DM individuals had a higher proportion of the dysbiotic *Bacteroides* 2 enterotype. No significant differences in microbial diversity were observed in T2DM individuals after controlling for cofounding factors, contrasting with reports from westernized cohorts. Interestingly, fungal diversity was significantly decreased in *Bacteroides* 2 enterotype. Functional profiling from 16 S rRNA gene data showed marked differences between T2DM and non-T2DM controls, with an enrichment in amino acid degradation and LPS-related modules in T2DM individuals, whereas non-T2DM controls had increased abundance of carbohydrate degradation modules in concordance with enterotype composition. These differences provide an insight into gut microbiome composition in Emirati population and its potential role in the development of diabetes mellitus.

## Introduction

The gut microbiome is a critical reservoir of microbial species and their genes and genomes present in the human gastrointestinal tract. Host genetics, environment, diet, the immune system, and many other lifestyle factors interact with the gut microbiome to regulate their composition and function^1^. Data are bringing convincing evidence that gut microbiome plays an important role in human health and diseases^2^. Studies have indeed linked gut microbiome richness and composition with a spectrum of cardiometabolic and neurodegenerative disorders including obesity, diabetes, cancer, depression, and schizophrenia amongst others^3–5^. Especially the pathogenic association between gut microbiome and type 2 diabetes is quickly gaining momentum in the world through many reports. This is also due to the availability of technological advancements in metagenomics, which enable the dissection of the complex relationship between gut microbiome and diabetes.

These reports suggested that T2DM is associated with dysbiosis, a reduction in microbiome richness, altered bacterial composition and functional properties^6^. Among these, were reported a lowered abundance of butyrate-producing microbes, an altered firmicutes / bacteroidetes ratio, and an increase in opportunistic pathogens, such as *Bacteroides caccae*, *Clostridium hathewayi*, *Clostridium ramosum*, *Clostridium symbiosum*, *Eggerthella lenta* and *E. coli*^3,7–10^. These changes may induce disturbances in host gut barrier, in metabolic homeostasis and low-grade inflammation, in short chain fatty acid synthesis and fat deposition as well as hormonal regulation for involving glucagon-like peptide-1 synthesis. These factors contribute to glucose metabolism alteration, insulin resistance and dyslipidemia in patients with diabetes^11–14^. While the interaction between gut microbiome and metabolic health has been studied in several populations, exploring these interactions in Middle East countries is of particular interest considering the very high prevalence of diabetes in this region of the world^15^. Researchers have mostly focused on examining the bacterial members of the gut microbiome, but very little is known about the fungal communities which are non-negligible components in the gut. Mycobiota have been described as members of the normal gut flora in 1967^16^. Fungal populations comprise less than 1% of the total gut microbiome. However, recent studies have indicated that these fungi have relevant effects on dampening inflammatory responses in the gut, especially in inflammatory bowel diseases despite their small amount^17,18^. Others have reported their impact on bacterial community composition^19–21^. Fungi may represents a key part of the microbial community with significant impact on the gut ecosystem, and possibly the host health^21^. However, the potential role of fungi and their interaction with the host and with other members of the gut community and metabolic health needs further understanding.

Research groups have demonstrated a significant impact of T2DM on gut microbial richness and relative abundance^4,22,23^ and underscored significant contribution of gut microbiome in T2DM phenotypes as insulin resistance and low-grade inflammation^24^. However, little is known about the relationships between T2DM on gut microbiome in UAE population. Here, we examined bacterial and fungal microbiome composition and possible functional consequences in T2DM individuals from an Emirati population. We performed 16 S rRNA gene and ITS2-based microbial profiling analysis of 50 stool samples from 25 T2DM and 25 non-T2DM individuals. We conducted a phylogenetic investigation of communities by reconstruction of unobserved states (PICRUSt) functional analyses based on 16 S rRNA gene abundance profiles to gain deeper insight on potential functional impact on the host in T2DM from this Emirati population.

## Materials and methods

### Patient inclusion and ethical statement

The study was performed after receiving the necessary ethical approval from University Hospital Sharjah Ethics Research Committee (UHS-HERC-021-0702). The study was performed in accordance with relevant research guidelines and regulations of the committe. We randomly identified 25 native Emirati subjects with diagnosis of T2DM attending the endocrinology clinic. We also identified 25 otherwise healthy Emirati individuals and had HbA1c level < 6% as controls. All volonteers were provided with information sheet and explanation of study objectives, design, and confidentiality. We obtained written informed consents. We provided to all subjects a sterile stool specimen container with integrated collection spoon and collection instructions. A total of 50 stool specimens, 2 to 4 grams of freshly passed stool was collected in sterile containers. The specimens were stored immediately in liquid nitrogen and transferred to −80°C for storage until further analysis. Liquid (diarrheal) stools and use of antibiotics in the last 3 months were the exclusion criteria for this study.

### DNA extraction

Faecal samples were subjected to DNA extraction using QIAamp PowerFecal DNA Kit (Qiagen Ltd, GmbH, Germany) following the manufacturer’s instruction (Qiagen Ltd). The extracted DNA was stored at −80°C for further analysis.

### Bacterial and fungal PCR, sequencing, and sequence analysis and Taxonomic composition

Bacterial 16 S rRNA genes were amplified using polymerase chain reaction (PCR) targeting the V4 region with dual-barcoded, as per procedure as described in^25^. Next, amplicons sequenced with an Illumina MiSeq using the 250-bp paired-end kit (v.2). Sequences were denoised, taxonomically classified using Greengenes (v. 13_8) as the reference database, and clustered into 97% similarity operational taxonomic units (OTUs) with the mothur software package (v. 1.39.5) previously described^26^, following the recommended procedure (https://www.mothur.org/wiki/MiSeq_SOP; accessed August 2018). The resulting dataset had 21257 OTUs (including those occurring once with a count of 1, or singletons). An average of 18383 quality-filtered reads generated per sample. Sequencing quality for R1 and R2 was determined using FastQC 0.11.5.

ITS2 region were sequenced on an Illumina MiSeq (v. 2 chemistry) using the dual barcoding protocol as described^25^. Primers and PCR conditions used for 16 S rRNA gene and ITS2 sequencing were identical to those previously described^27^. Bacterial sequences were processed and clustered into operational taxonomic units (OTUs) with the mothur software package (v. 1.39.5)^26^, following the recommended mothur SOP. Paired-end reads were merged and curated to reduce sequencing error as described in^28^. The resulting dataset had 3171 OTUs (including those occurring once with a count of 1, or singletons). An average of 9581 quality-filtered reads were generated per sample. Sequencing quality for R1 and R2 was determined using FastQC 0.11.5. Fungal processing pipeline was identical as the one used for bacteria, except for the following differences: (1) paired-end reads were trimmed at the non-overlapping ends, and (2) high quality reads were classified using UNITE (v. 7.1) as described before as the reference database^29^. A consensus taxonomy for each OTU obtained and the OTU abundances then aggregated into genera. OTU table was rarified to 10000 reads per sample to correct for differences in sequencing depth with *rarefy_even_depth function* of *phyloseq* R package^30^, and alpha diversity indexes (Observed species, Shannon, ACE) were computed from rarified OTU table *estimate_richness* function of *phyloseq* R package. The R package *vegan* was used to compute Beta-diversity matrix from rarified OTU table collapsed at genus level (*vegdist* function) and to visualize microbiome similaritires with principle coordinate analysis (PCoA) (*cmdscale* function)^31^. Enterotype classification was performed from the same genus abundance matrix used for PCoA analyses following two different approaches. First, samples were clustered using Jensen-Shannon divergence (JSD) distance and the Partition Around Medoids (PAM) clustering algorithm as described in Aurumugam *et al*^32^. Second, samples were clustered from genus abundance data using the Dirichlet Multinomial Mixture (DMM) method of Holmes *et al*^33^. The DMM approach groups samples if their taxon abundances can be modeled by the same Dirichlet-Multinomial (DM) distribution.

### Quality control

The possibility for contamination examined by co-sequencing DNA amplified from samples and from four each of template-free controls and extraction kit reagents treated the same way as the samples. Two positive controls, consisting of cloned SUP05 DNA, were also added (number of copies = 2*10^6). Operational taxonomic units were considered putative contaminants (and were removed) if their mean abundance in controls reached or surpassed 25% of their mean abundance in samples as described before^34^.

### Functional profiling from 16 S rRNA gene data

Gene family abundances from Kegg Orthology (KO) functional space were computed from rarified 16 S rRNA gene OTU abundance matrix and GreenGenes taxonomic annotations with PICRUSt-1.1.3^35^. This includes correction of OTU abundances by 16 copy number of reference GreenGenes taxons with *normalize_by_copy_number.py* script, compute KO abundance matrix from 16 S rRNA gene copy number-corrected 16 S rRNA gene OTU abundance matrix with *predict_metagenomes.py* script, and determine OTU contributions to each KO abundance vector with *metagenome_contributions.py* script. Gut Metabolic Modules (GMMs) were quantified from the PICRUSt KO abundance matrix with *GOmixer* R package^36^.

### Statistical analysis

Linear regression analyses was used to evaluate the impact of different clinical variables (age, BMI, weight, diet and gender) and disease state over alpha diversity distribution. The significance of diversity changes after excluding the variability explained by age cofounder was tested with non-parametric Wilcoxon test over the residuals of linear regression analyses of alpha diversity (dependent variable) vs. age (independent variable). To evaluate beta diversity across samples, we excluded genus occurring in fewer than 10% of the samples with a count of less than three and calculated Bray-Curtis indices. Environmental fitting of clinical variables (age, BMI, weight, diet and gender) and disease state over Principal coordinates analyses ordination from Bray-Curtis inter-sample dissimilarity matrix was computed with *envfit* and *cmdscale* functions of vegan R package^37^. Dissimilarity in community structure by disease state was assessed with permutational multivariate analyses of variance (PERMANOVA) with non-T2DM *v.s* T2DM groups as the main fixed factor and using 4,999 permutations for significance testing with *adonis* function of *vegan* R package.

To identify taxonomic and functional features associated to disease state while accounting for cofounding effect of age generalized linear models (GLM) with negative binomial distribution were fitted with feature abundance as dependent variable and disease state and age as dependent variables with DESEq. 2^38^ and Phyloseq.^30^ R packages. Functional enrichment analyses of KEGG modules were carried out to identify high-order functional features associated to T2DM transition from KO adjusted P-values and log2 fold changes between health controls and T2DM as effect sizes using the Reporter Feature algorithm as implemented in the *Piano* R package^39^. The null distribution was used as significance method and P-values were adjusted for multiple comparisons with the Benjamini-Hochberg method^40^. All analyses were conducted in the R environment.

## Results

### Gut microbiome profile of T2DM Emirati subjects: compositional differences between non-T2DM and T2DM subjects

We evaluated the intra- and inter-individual variability of gut microbiome among 25 T2DM and 25 non-T2DM subjects, all from Emirati origin. Their clinical characteristics are shown in S1 Table. T2DM subjects were significantly older, had higher BMI and were more sedentary than non-T2DM subjects were (P value < 0.05; Table S1). Further, based on short food frequency questionnaire (DFI-FFQ)^41^, we found higher percentage of T2DM individuals with a high fiber diet compared to non-T2DM individuals (P value < 0.05; Table S1). All T2DM individuals were under Dipeptidyl peptidase-4 inhibitors (DPP4i) and metformin treatment.

### S1 Table: Clinical characteristics of the study groups

Median and quartiles 1 and 3 are shown for continuous variables. Number and percentage of samples are shown for categorical variables. P values are computed from Wilcoxon rank-sum test for continuous variables and chi-squared or exact Fisher test when the expected frequencies is less than 5 in some cell. False discover rate (FDR) were computed with Benjamini-Hochberg method.

**Figure 1:**
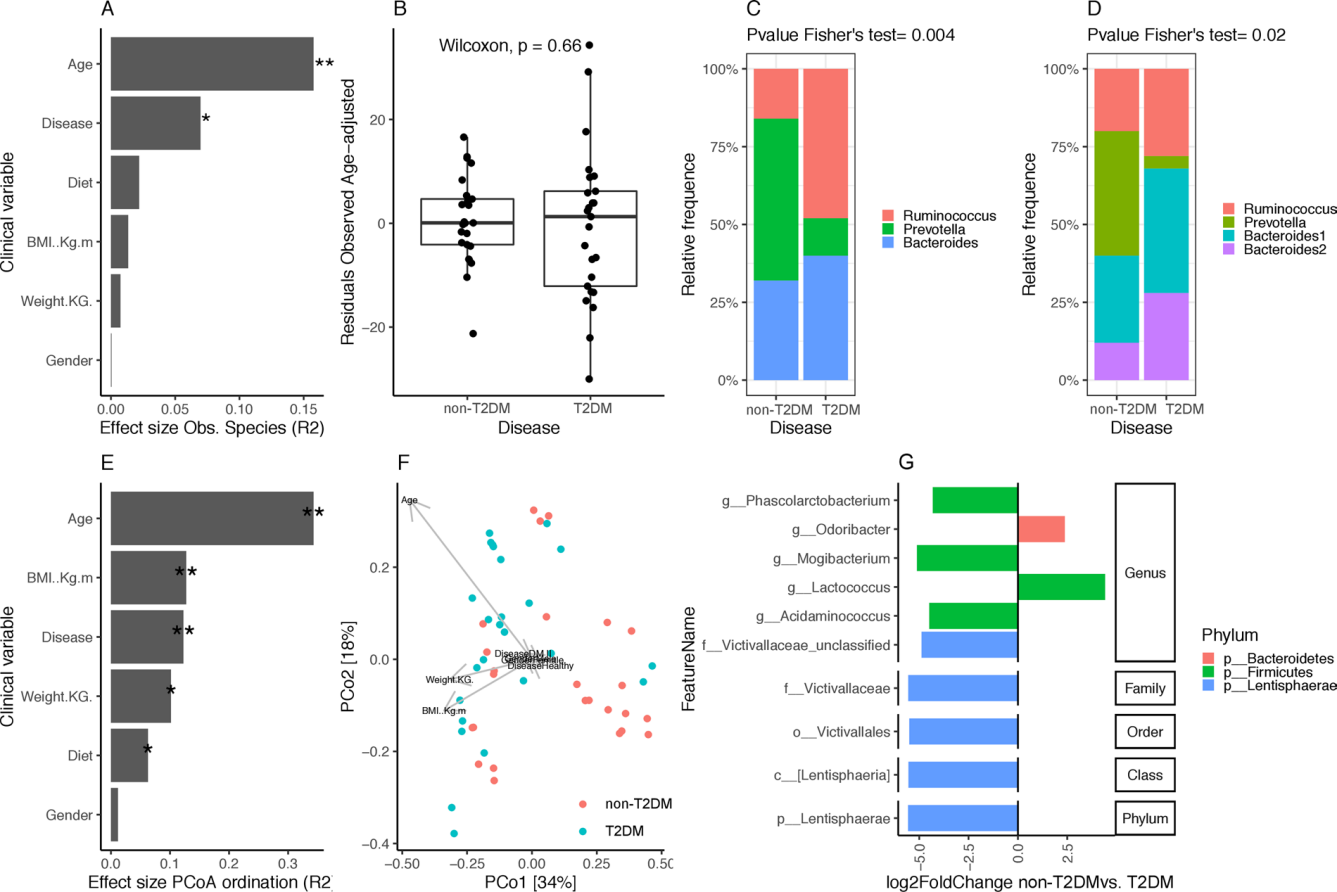
Prokaryotic profiling of gut microbiome. (**A**) Effect sizes of clinical covariates and disease state over Alpha diversity distribution (observed species) based on linear regression analyses (** = FDR < 0.05; * = P value < 0.05, FDR > 0.05) (**B**) Differences in residuals of linear-regression between alpha diversity (Observed species, dependent variable) and age (independent variable) between study groups. (**C**) Enterotype composition in non-T2DM and T2DM individuals by PAM clustering over JSD distance matrix computed from genus abundance data. (**D**) Enterotype composition in non-T2DM and T2DM individuals by DMM approach from genus abundance data. (**E**) Effect sizes of environmental fitting of clinical variables and disease state over PCoA ordination (** = P value < 0.05; * = P value < 0.1; permutation test) (**F**) Principal coordinates analyses of inter-individual differences (genus-level Bray-Curtis beta-diversity) with samples colored by disease state (non-T2DM, T2DM). Arrows represents effect sizes of the significant variables identified by environmental fitting analyses of panel E. (**G**) Barplot of log2 fold changes in taxonomic feature abundances between health controls and T2DM (P value < 0.05 in GLM model with negative binomial distribution of feature abundance by disease state adjusted by age).

Linear regression analyses of individual covariates (age, diet, BMI, weight, and gender) and disease state over alpha diversity (observed species) shows that age has an important effect over microbiome diversity (p value < 0.05; R2 = 0.16), with alpha diversity levels significantly increasing with age (Spearman Rho = 0.4; P value < 0.05) (Fig. 1A). When we take out the variability explained by age no significant differences in microbial diversity were observed between non-T2DM and T2DM individuals (Fig. 1B; Wicoxon rank-sum test on the residuals of linear regression analyses of observed species by age; P value = 0.66), with a wider variability in microbiome diversity observed in the T2DM group. Similar results were observed with other alpha diversity indexes (ACE, Shannon; Supplemental Fig. 1A–D).

We further examined the gut microbiota characteristics in terms of community composition. Sample clustering based on genus-level 16 S rRNA gene abundance data shows the presence of microbial enterotypes that characterize gut microbiome composition in European, Asian and American cohorts^42^. PAM clustering of samples from JSD beta diversity matrix at k = 3 shows the presence of *Bacteroides*, *Ruminococcus* and *Prevotella* enterotypes according to the abundance distribution of these prokaryotic genera (Supplemental Fig. 2A,D–F). DMM clustering with genus abundance matrix splits *Bacteroides* enterotype into two subgroups (Supplemental Fig. 2B) as previously described^43^ (*Bacteroides*_1 and *Bacteroides*_2^43^, after additional re-assignments of *Prevotella* samples to *Ruminococcus* (n = 3) and *Bacteroides*_1 (n = 2) and *Ruminococcus* samples to *Bacteroides*_1 enterotype (n = 7) (Supplemental Fig. 2C).

Diversity distributions across these enterotypes confirm in this Emirati population with the high diversity profile associated with *Ruminococcus* enterotype and the low diversity profile associated with *Bacteroides* 2 enterotype (Supplemental Fig. 3). Further, T2D and non-T2D groups show significant differences in microbiome composition according to different enterotyping methods. PAM clustering over JSD beta diversity matrix shows that the non-T2D group is enriched in Prevotella enterotype, whereas the T2D group is enriched in Ruminococcus enterotype (Fig. 1C, Fisher’s exact test < 0.05). When enterotyping is carried out with the Dirichlet Multinomial Mixture method, we still observe that non-T2D controls are enriched in Prevotella enterotype, whereas an enrichment of the low-diversity Bacteroides2 enterotypes is observed in the T2D group (Fig. 1D, Fisher’s exact test < 0.05). We also observed that 7 Ruminococcus samples with PAM clustering has been re-assigned to Bacteroides1 enterotype with the DMM method (Supplemental Fig. 2C), a dysbiotic microbiome composition associated to low microbial cell density and enriched in Crohn and IBD^43,44^. Environmental fitting of disease and other covariates over PCoA ordination space from genus abundance matrix shows a significant impact of disease over microbiome composition (R2 = 0.12; P value = 0.001) together with age (R2 = 0.34, P value = 0.001) and BMI (R2 = 0.13, P value = 0.037) (Fig. 1E,F).

Finally, we search for taxonomic features significantly different between non-T2DM and T2DM groups while accounting for cofounding variables detected in environmental fitting analyses by fitting generalized linear models of genus abundance by disease, age and BMI with negative binomial distribution from raw abundance feature counts with DESeq2^38^. Six bacterial genus were significantly associated to disease state (P value < 0.05), four of them increased in T2DM group (*Phascolarctobacterium, Mogibacterium, Acidaminococcus* and Unclassified *Victivallaceae*; log2 fold change Health vs. T2DM < 0), whereas two of them were decreased in T2DM group (*Odoribacter* and *Lactococcus;* log2 fold change non-T2DM vs. T2DM > 0) (Fig. 1G). The association with Unclassified *Victivallaceae* is reproduced at higher taxonomic levels (from family to phylum; Fig. 1G). None of these features resist P value adjustment by multiple comparisons (FDR > 0.05).

### Fungal composition is different between T2DM and non-T2DM subjects

**Figure 2:**
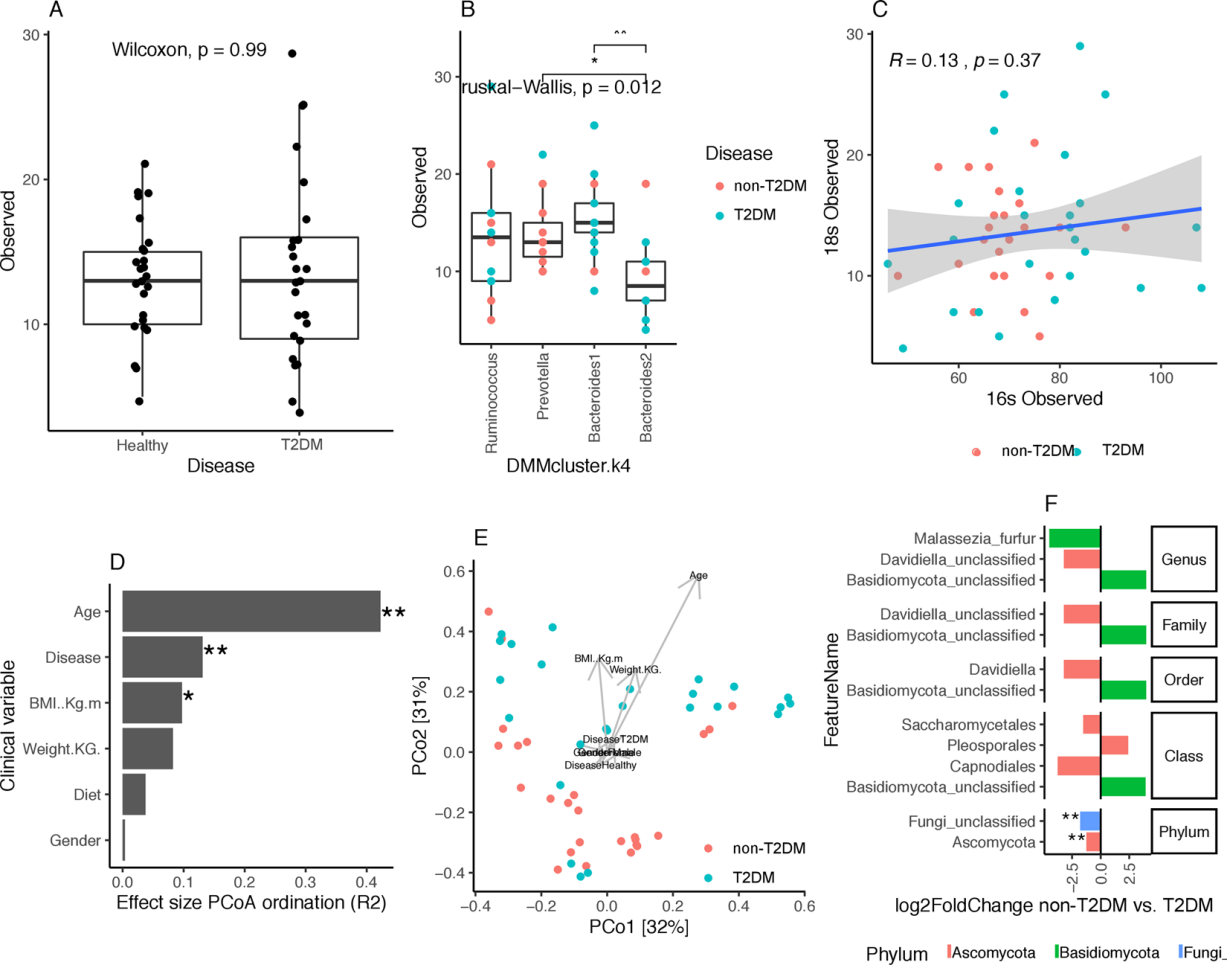
Fungal profiling of gut microbiome. (**A**) Alpha diversity distributions (observed species) between non-T2DM and T2DM groups. (**B**) Fungal diversity distributions (observed species) across DMM enterotypes (** = P value < 0.001; * = P value < 0.05; Wilcoxon rank-sum test). (**C**) Correlation between fungal and prokaryotic diversity (observed species). R and p corresponds to Spearman Rho and p-value of Spearman correlation test. (**D**) Effect sizes of environmental fitting of clinical variables and disease state over Principal coordinates ordination from panel E (** = P value < 0.05; * = P value < 0.1; permutation test). (**E**) Principal coordinates analyses of inter-individual differences (genus-level Bray-Curtis beta-diversity) with samples colored by disease state (non-T2DM, T2DM). Arrows represents effect sizes of the significant variables identified by environmental fitting analyses of panel D. (**F**) Bar plot of log2 fold changes in taxonomic feature abundance between non-T2DM controls and T2DM (P value < 0.05 in GLM model with negative binomial distribution of feature abundance by disease state adjusted by age).

Fungi comprise a small percentage of the gut microbiome^16^, but reports have indicated that fungi have surprisingly strong effects on dampening inflammatory responses in the gut^17,18^. Others reported fungi impact on bacterial community composition^19,20^. Here, using ITS profiling we observed no significant difference in fungal diversity between T2DM and non-T2DM controls (P-value > 0.05 Wilcoxon test, Fig. 2A). In contrast with what we observed with prokaryotic diversity, linear regression analyses of individual covariates (age, diet, BMI, weight and gender) shows no significant associations of any of them with fungal diversity (P value > 0.05; Supplemental Fig. 4). We found no significant association between fungal and prokaryotic diversity (rho = 0.13; p value > 0.05, Fig. 2C). However, relating fungal diversity with enterotype composition, we found significant differences in fungal diversity across DMM enterotypes (P-value < 0.05 Kruskal-Wallis test; Fig. 2B), with *Bacteroides* 2 enterotype showing significant lower levels of fungal diversity in comparison with *Bacteroides* 1 and *Prevotella* groups (Fig. 2B).

Next, we examined the fungal microbiome composition as previously performed for bacterial composition. Environmental fitting of disease and other covariates over PCoA ordination space from fungal genus abundance matrix shows age (R2 = 0.42, P value = 0.001) and disease (R2 = 0.13, P value = 0.001) as the main variables with significant impact over fungal microbiome composition (Fig. 2D–E). In order to find fungal features associated to disease state while taking into account the confounding effect of age detected by environmental fitting, we follow the same approach as described above for 16 S rRNA gene data (fit generalized linear models of fungal feature abundance by disease and age with negative binomial distribution from raw feature counts). We observe a significant association of three fungal genenera with disease state (P value < 0.05), two of them (*Malessezia firfur* and Unclassified *Davidiella*) increased in the T2DM group (log2 fold change non-T2DM vs. T2DM < 0) and one (Unclassified *Basidiomycota*) decreased in the T2DM group (log2 fold change non-T2DM vs. T2DM > 0) (Fig. 2F). At higher taxonomic levels, T2DM groups seems to be characterized by an increase of *Ascomycota* lineages and a decrease of unclassified *Basidiomycota* lineages (Fig. 2D).

### Functional profiling of T2DM and non-T2DM groups microbiomes based on 16 S rRNA gene profiles

**Figure 3:**
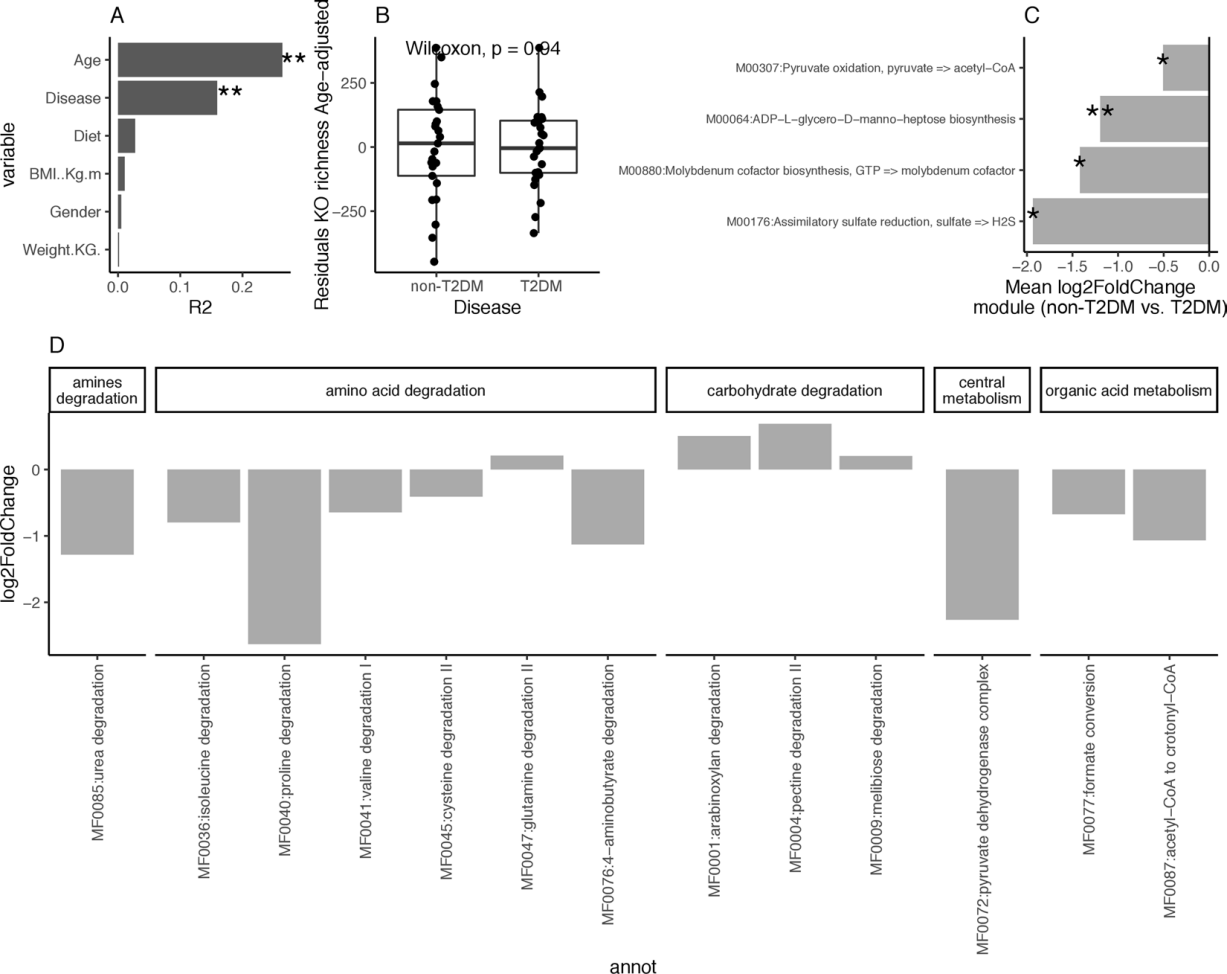
Functional profiling based on PICRUS analyses of 16 S data. (**A**) Effect sizes of clinical covariates and disease state over functional diversity distribution (KEGG orthology (KO) groups identified in PICRUSt analyses) based on linear regression analyses (** = FDR < 0.05; * = P value < 0.05, FDR > 0.05). (**B**) Differences in residuals of linear-regression between functional diversity (Observed KOs, dependent variable) and age (independent variable) between study groups. (**C**) KEGG modules significantly enriched in differentially abundant KO groups between non-T2DM and T2DM group (** FDR < 0.05, * = P value < 0.05; Gene Set Enrichment Analyses). The mean log2 fold changes of module KOs abundances between non-T2DM controls and T2DM is represented as indicator of enrichment direction (all modules enriched in the T2DM group; mean log2 fold changes non-T2DM controls vs. T2DM < 0). (**D**) Bar plot of log2 fold changes in Gut metabolic modules (GMMs) abundances between health controls and T2DM (P value < 0.05 in GLM model with negative binomial distribution of GMM abundance by disease state adjusted by age).

We used the PICRUSt tool to project the functional content of the prokaryotic microbiome in the studied samples from 16 S rRNA gene OTU abundance data. In agreement with taxonomy findings, linear regression analyses of individual covariates (age, diet, BMI, weight, and gender) and disease state over functional diversity (observed KO groups) shows that disease (R2 = 0.16, P value < 0.05) and age (R2 = 0.26, P value < 0.001) have a significant impact over functional diversity (Fig. 3A). Functional diversity levels significantly increase with age (Spearman Rho = 0.51; P value < 0.001). When we excluded the variability explained by age no significant differences in functional diversity were observed between non-T2DM and T2DM individuals (Fig. 3B; P value = 0.94; Wicoxon rank-sum test on the residuals of linear regression analyses of observed species by age). Environmental fitting of disease and other covariates over PCoA ordination space from KO abundance matrix shows weight (R2 = 0.35, P value = 0.002), age (R2 = 0.29, p value = 0.001), BMI (R2 = 0.24, p value = 0.001), disease (R2 = 0.17, p value = 0.002) and diet (R2 = 0.07, p value = 0.033) as the variables with significant impact over functional prokaryotic content of the gut microbiome (Supplemental Fig. 5).

Generalized linear models with negative binomial distribution of KO raw count data by disease state adjusted by age (4129 KOs with at least 10 counts in >20% of the samples) showed 210 KO groups significantly associated to disease state (FDR < 0.05), 32 decreased in the T2DM group (log2 fold change non-T2DM vs. T2DM group > 0) and 178 increased in the T2DM group (log2 fold change non-T2DM vs. T2DM group < 0). In order to find higher-level functional associations, we used gene set enrichment analyses of KEGG functional modules with adjusted p-values from age-adjusted GLM models and log2 fold changes of KO abundances of non-T2DM vs. T2DM as indicators of effect size. Four KEGG modules were significantly enriched in differentially abundant KOs (p value < 0.05), all of them enriched in KOs significantly increased in T2DM group (mean module KO log2 fold changes health vs. T2DM < 0). Among these we found M00064 (ADP-L-glycero-D-manno-heptose biosynthesis), a module representing the biosynthesis of glycero-manno-heptoses found in the lipopolysaccharides (LPS) of most Gram-negative bacteria, capsules and O-antigens of some Gram-negatives, and in the S-layer of certain Gram-positive bacteria^45^. Also we observed an enrichment of M00176 (assimilatory sulfate reduction), which was previously identified as signature of T2DM^9^, and an enrichment of pyruvate oxidation module (M00307) representing the pyruvate dehydrogenase complex, a key enzymatic complex linking glycolysis to TCA cycle in central metabolism during aerobic respiration^46^. Finally, quantification of Gut Metabolic Modules (GMM)^47^ based on KO abundance data shows 14 GMMs associated to disease state (Fig. 3D; FDR < 0.05; GLM models based on negative binomial distribution of module abundance by disease state adjusted by age). This analyses shows marked differences in the functional profile of gut microbiome of T2DM and non-T2DM controls, with non-T2DM controls showing significantly increases in different carbohydrate degradation modules (arabinoxylan, pectine and melibiose degradation modules, log2 fold change non-T2DM vs. T2DM > 0), whereas T2DM group showing significantly increases in several aminoacid degradation modules (isoleucine, proline, valine, cysteine, glutamine and aminobutyrate; log2 fold change non-T2DM vs. T2DM < 0), confirming also the increases in pyruvate dehydrogenase compex in T2DM group observed in the KEGG module enrichment analyses (Fig. 3C,D).

## Discussion

In this study, we characterized for the first time, the prokaryotic and fungal microbiome profiles associated with T2DM and non-T2DM controls in an Emirati population where the study population was unmatched for age, BMI, and diet. When we evaluated the impact of these covariates together with disease state on microbiome diversity and composition, we observed that age had an important effect over microbiome diversity and composition. However, when we adjusted for age, there were no significant differences in microbial diversity between non-T2DM and T2DM controls. Remarkably and in contrasts with results of previous studies in westernized populations, where several factors impact gut microbiome composition and can be seen as confounders such as dietary habits, lifestyle and age^48–53^. One explanation can be related to dietary factors that are known to strongly impact gut microbiome composition^54^. For example, an Australian group demonstrated a significant effects of nutritional counseling on gut microbiome abundance and diversity among T2DM and obese individuals^55^. In our study, all T2DM individuals were subjected to rigorous dietary counselling as part of their clinical follow-up with a nutritionist. Furher, dietary aspects may contribute to some genera enrichment. For example, it is well known that fibers impact on *Prevotella* abundance which aids in polysaccharide breakdown^56,57^. In our study, we noticed an enrichment in *Prevotella* in the non-T2DM controls despite lower fiber intake based on the DFI-FFQ evaluation (Table S1). This observation is consistent with significant increase in carbohydrate degradation modules observed in the GMM modules analyses. Further, we detected an increase in aminoacid degradation modules in the T2DM group, which is in line with the observed enrichment of *Bacteroides* 2 enterotype and the proteolytic character of *Bacteroides* group^58^. Moreover, among the taxonomic features that resist age adjustment, we reported an increase of *Victivallaceae* lineage belonging to *Lentisphaera* phylum in the T2DM group and was notably identified from genus to phylum level. This lineage has been associated with gestational diabetes melitus in children^59^ and has been described to significantly increase in individuals consuming gluten-free diet^60^, again suggesting a potential association with the dietary counseling among T2DM group. The genus *Phascolarctobacterium* has also been associated both positively^61–63^ and negatively^64^ with markers of insulin sensitivity, whereas the genus *Odoribacter*, which includes butyrate producing bacteria that has been described negatively associated with hypertension in obese pregnant woman^65^. This genus also decreases in response to pre-natal metformin exposure in mice experiments^66^. *Acidaminococcus* genera has been also associated with modestly lower risk of T2DM in a mendelian randomization study^67^. However, the particularities of our study cohort in terms of ethnicity, and age and nutritional counseling between groups makes it difficult to extrapolate additional conclusions without further experimental evidences. All together, these findings underscores an important contribution of dietary counselling in driving these compositional changes^68^.

Another explanation to the observed difference from previous studies in westernized populations can be related to metformin administration among all T2DM subjects. We observed increased releative abundance of *Escherichia*, *Akkermansia muciniphila* and other unclassified *Enterobacteriales* lineage in T2DM subjects receiving metformin treatment. However, these differences do not resist adjustment by age. The increase in *Escherichia coli* and *A.muciniphila* in T2DM have been repeatedly reported in literature, and often associated with metformin intake^69,70^.

Next, we determined the presence of enterotypes that characterize microbiome composition. *Prevotella* enterotype is enriched in non-T2DM control group and *Ruminococcus* and *Bacteroides* 2 enterotypes is enriched in T2DM group. The compositional profile of T2DM group was also found to be heterogeneous, with enrichment of *Ruminococcus* enterotype that is usually associated with a more diverse microbiome profile^32^ and *Bacteroides* 2 enterotype, which generally shows an opposite association, being characterized by low microbial diversity and microbial loads and enriched in Crohn’s disease and ulcerative colitis patients^43^. This is also reflected in the wider range of prokaryotic diversity observed in the T2DM group in comparison with non-T2DM controls indicating a more heterogeneous microbiome profile in T2DM group, that could be attributed again to lifestyle habits as well as differences in T2DM severity.

The definition of discrete community types is a challenging task given the complexity in the landscape of community composition existing in the gut microbiome and the wide within and between-individual diversity existing in the human’s gut, which makes difficult extrapolation of conclusions based on discrete clusters to individuals in the boundary of different groups^71,72^. Also, and more importantly, sample clustering is strongly dependent of the other samples analyzed at the same time, which makes discretization dependent of the compositional landscape of the analyzed cohort, difficulty comparisons across studies. However, multiple studies have reproduced the presence of enterotypes with similar compositional properties across large datasets from different origins^42^, and the split of *Bacteroides* groups into two subgroups with the DMM method and the dysbiotic profile of the *Bacteroides* 2 group has been reproduced also in different studies and cohorts^43,44,73,74^. Thereby, a larger cohorts would be necessary to evaluate the strength of these community types across the Emirati population or if alternative community types could be defined.

Finally, we explored the gut microbiome functional contribution. Interestingly and in spite of the cofounding effects of age, we still observed signals at the functional level that have been identified in other quantitative metagenomic studies of T2DM, suggesting a more inflammatory profile in T2DM individuals^53^. For example, we noted an enrichment of ADP-L-glycero-D-manno-heptose biosynthesis module in T2DM group, a component of the bacterial LPS, associated with T2DM individuals and in agreement with other studies^69^. This molecule corresponds to one of the most antigenic part of the LPS, associated with low-grade inflammation that usually take place in obesity and T2DM^69,75^. In addition, it has been recently demonstrated as a potent pathogen-associated molecular pattern (PAMP) recognized by ALPK1 receptor and iducing NF-κB activation and cytokine expression^76^. Additionally, the formate conversion GMM significantly increased in the T2DM group (Fig. 3D) corresponding to the formate dehydrogenase complex responsible for formate oxidation, a metabolic signature of a dysbiosis-induced intestinal inflammation^77^.

Regarding fungal microbiome effect, we observed no significant differences in fungal diversity between T2DM and non-T2DM subjects. However, we detected a significant impact of disease state over fungal microbiome composition, even after normalizing the confounding impact of age. Remarkably, we found that *Bacteroides* 2 enterotype was associated with decreased levels of fungal diversity, in addition to its known dysbiotic phenotype, in terms of microbial diversity and loads in different pathologies like IBD and UC^43^. This observation extends previous findings showing that the deleterious B2 enterotype also associates with a decrease in fungal diversity. Thus, fungal diversity might been seen as an additional and novel signature of this dysbiotic microbiome composition that would need further validation in larger cohorts with fungal metagenomic data. Furthmore, we observed a shift from *Candida albicans* (known opportunistic) to *Candida glabrata* in the T2DM patients. Presence of *C. glabrata* has been linked to supressing genes involved in mannan biosynthesis, an important component of fungal cell wall with known protective benefits to the host^78,79^. Whether, this compositional shift from known commensal fungi to their virulent counterparts and the dissimilarity in mannan biosynthesis, significantly alters the intestinal barrier is yet to be explored.

In conclusion, we report a shift in gut microbiome composition and function among individuals affected by T2DM as compared to non-T2DM controls in a pilot study of Emirati people. The study population was distinctively unmatched for age, BMI, and diet, thereby providing a unique pattern and more challenging approach. Gut microbiome peculiarities have been linked to T2DM across the globe based on variation in diet, medication and ethnicity among other factors. Remarkably, our study revealed no significant differences in taxonomic and functional diversity among T2DM group, in contrast to what has been reported elsewhere, but we observed significant differences in microbiome composition (enterotypes) and functional content between study groups despite the added complexity by the unmatched confounders. We recognize that our results can be influenced by the divergence in mean age, diet intervention and highly individualized gut microbiome composition. We attributed these differences to dietary counselling provided to T2DM patients. Further, we showed that the enterotype B2 appears linked to fungal diversity that could be an additional and novel signature of this dysbiotic microbiome. We acknowledge potential limitations of this study, including relatively small sample size, detailed information regarding lifestyle and more advanced functional analysis. However, despite these limitations, this study provides meaningful insight into links between gut microbiome and its fungal community in T2DM subjects in native Emirati people. These aspects will be important to understand functional role of gut microbiome and its alterations to support host-homeostasis against metabolic and inflammatory disorders.

## Supplementary information

Supplementary information.

Supplementary information 2.

## Data Availability

Sequencing data have been deposited in the European Bioinformatics Institute (EBI) European Nucleotide Archive (ENA) under accession number XXXX (Private access until paper acceptance). All other data generated or analyzed during this study are included in this published article (and its Supplementary Information files).
