# Publisher Correction: Revealing links between gut microbiome and its fungal community in Type 2 Diabetes Mellitus among Emirati subjects: A pilot study

**DOI:** 10.1038/s41598-021-85464-3

**Published:** 2021-03-11

**Authors:** Mohammad Tahseen Al Bataineh, Nihar Ranjan Dash, Pierre Bel Lassen, Bayan Hassan Banimfreg, Aml Mohamed Nada, Eugeni Belda, Karine Clément

**Affiliations:** 1 College of Medicine, University of Sharjah, Sharjah, United Arab Emirates; 2 Research Institute for Medical & Health Sciences At University of Sharjah, Sharjah, United Arab Emirates; 3 Sorbonne University, INSERM, Nutrition and Obesities: Systemics Approaches (NutrOmics), Research Unit, Assistance Publique Hôpitaux de Paris, Nutrition Department, Pitié-Salpêtrière Hospital, Paris, France; 4 College of Engineering, American University of Sharjah, Sharjah, United Arab Emirates; 5 University Hospital Sharjah, Sharjah, United Arab Emirates; 6 Integromics. Institute of Cardiometabolism and Nutrition (ICAN), Paris, France

Correction to: *Scientific Reports* https://doi.org/10.1038/s41598-020-66598-2, published online 15 June 2020

In the original version of this Article, the author Pierre Bel Lassen was incorrectly indexed. This error has now been corrected.

