# Author Correction: Revealing links between gut microbiome and its fungal community in Type 2 Diabetes Mellitus among Emirati subjects: A pilot study

**DOI:** 10.1038/s41598-021-94439-3

**Published:** 2021-07-19

**Authors:** Mohammad Tahseen Al Bataineh, Nihar Ranjan Dash, Pierre Bel Lassen, Bayan Hassan Banimfreg, Aml Mohamed Nada, Eugeni Belda, Karine Clément

**Affiliations:** 1 College of Medicine, University of Sharjah, Sharjah, United Arab Emirates; 2 Research Institute for Medical & Health Sciences at University of Sharjah, Sharjah, United Arab Emirates; 3 Sorbonne University, INSERM, Nutrition and Obesities: Systemics Approaches (NutrOmics), Research Unit, Assistance Publique Hôpitaux de Paris, Nutrition department, Pitié-Salpêtrière Hospital, Paris, France; 4 College of Engineering, American University of Sharjah, Sharjah, United Arab Emirates; 5 University Hospital Sharjah, Sharjah, United Arab Emirates; 6 Mansoura University, Mansoura, Egypt; 7 Integromics. Institute of Cardiometabolism and Nutrition (ICAN), Paris, France

Correction to: *Scientific Reports* 10.1038/s41598-020-66598-2, published online 15 June 2020

In the original version of this Article, an affiliation for author Aml Mohamed Nada was omitted. The additional affiliation is listed below:

Mansoura University, Mansoura, Egypt.

As a result, Affiliation 7 was incorrectly listed as Affiliations 6.

The original Article has been corrected.

