# Supplementary material for: Revealing links between gut microbiome and its fungal community in Type 2 Diabetes Mellitus among Emirati subjects: A pilot study
Source: Sci Rep. 2020 Jun 15;10:9624. doi: 10.1038/s41598-020-66598-2 (PMC7295773; doi:10.1038/s41598-020-66598-2)
Supplement: Supplementary file 1 — Supplementary information. [file 41598_2020_66598_MOESM1_ESM.pdf]

Revealing links between gut microbiome and its fungal community in Type 2 Diabetes Mellitus among Emirati subjects: A pilot study

Mohammad Tahseen AL Bataineh[1,2]*, Nihar Ranjan Dash[1], Pierre Bel Lassen[3], Bayan Hassan Banimfreg[4], Aml Mohamed Nada[5], Eugeni Belda[6], Karine Clément[3]*

Supplementary information

Additional files

Figure S1. ACE and Shannon diversity profiles by disease state

Figure S2. Enterotyping of study cohort.

Figure S3. Prokaryotic diversity distributions across enterotype calls.

Figure S4. Effect sizes of clinical covariates and disease state over fungal diversity (observed species) based on linear regression analyses.

Figure S5. Impact of study covariates over microbiome functional content from PICRUSt results.

Table S1. Clinical characteristics of the study groups.

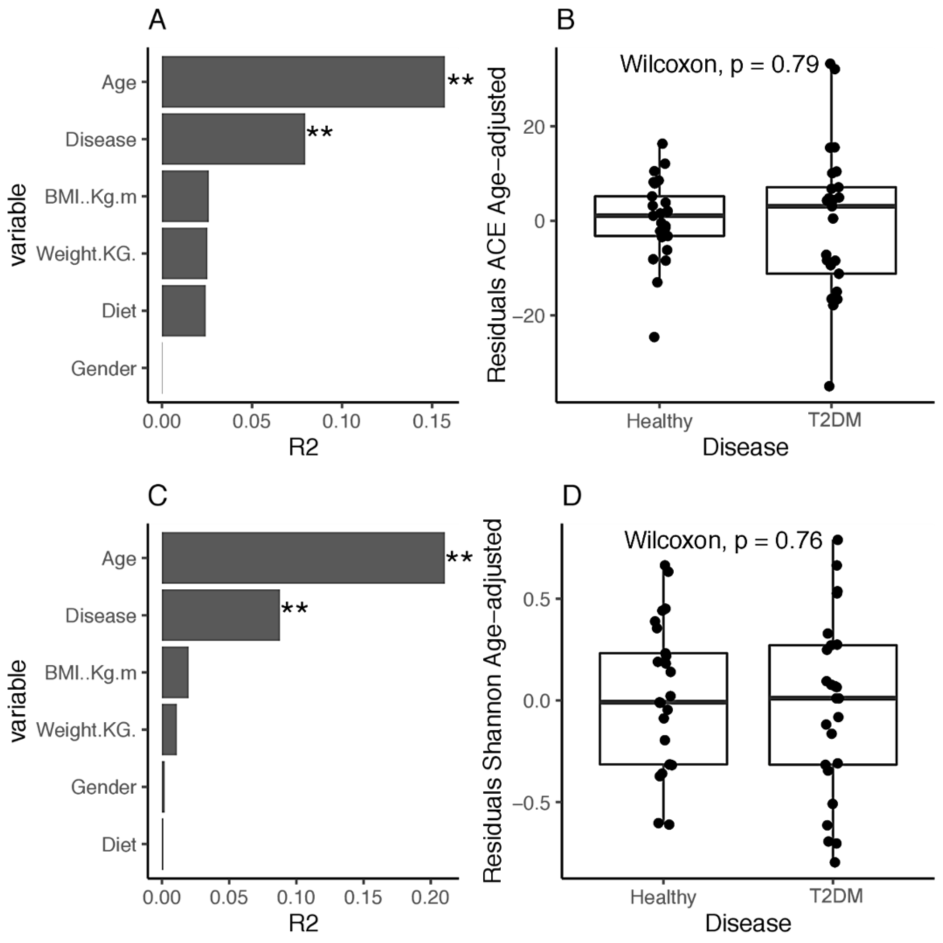
A
Age
Disease
BMI..Kg.m
Weight.KG.
Diet
Gender
variable
**
**
0.00
0.05
0.10
0.15
R2
B
Wilcoxon, p = 0.79
Residuals ACE Age−adjusted
20
0
−20
Healthy
T2DM
Disease
C
Age
Disease
BMI..Kg.m
Weight.KG.
Gender
Diet
variable
**
**
0.00
0.05
0.10
0.15
0.20
R2
D
Wilcoxon, p = 0.76
Residuals Shannon Age−adjusted
0.5
0.0
−0.5
Healthy
T2DM
Disease

Figure S1

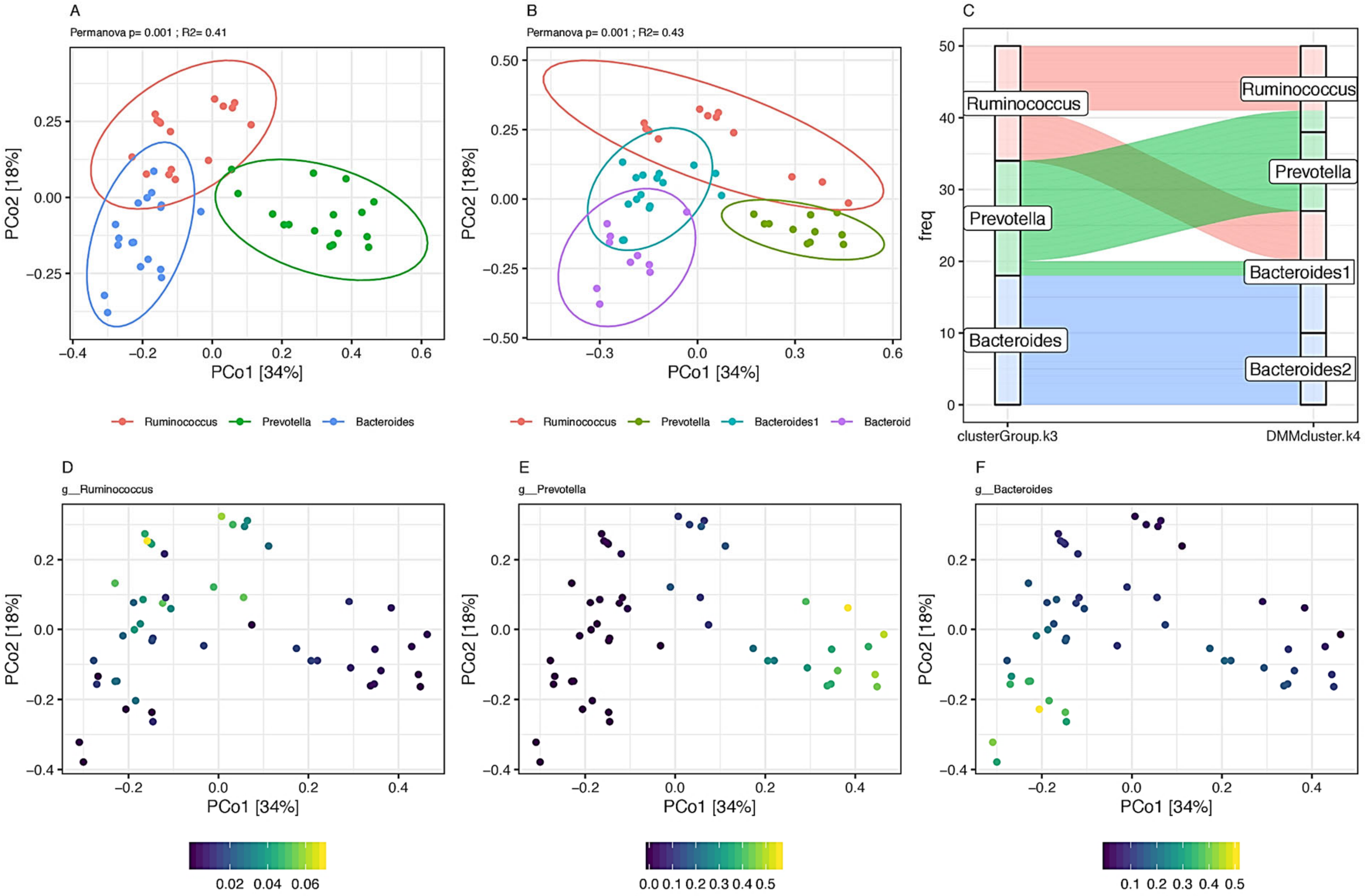
A
Permanova p= 0.001 ; R2= 0.41
B
Permanova p= 0.001 ; R2= 0.43
C
PCo1 [34%]
PCo2 [18%]
Ruminococcus
Prevotella
Bacteroides
Bacteroides1
Bacteroid
freq
clusterGroup.k3
DMMcluster.k4
Bacteroides2
D
g__Ruminococcus
E
g__Prevotella
F
g__Bacteroides

Figure S2

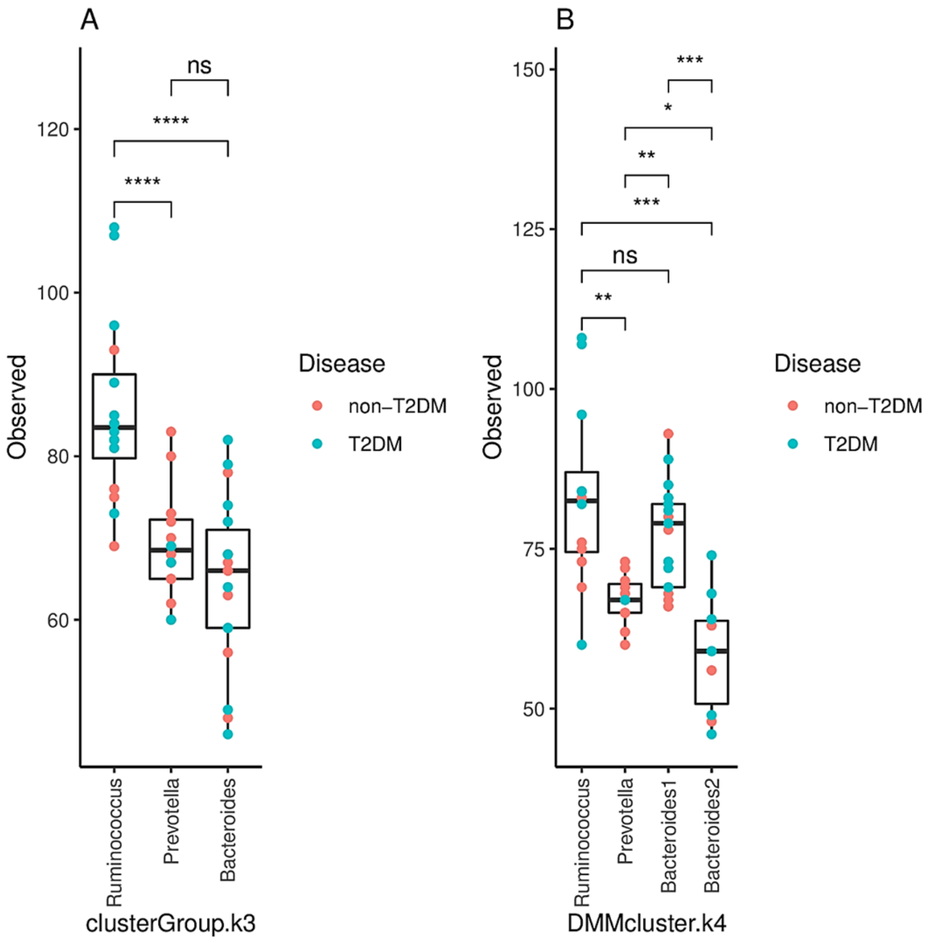
A
ns
****
****
120
100
80
60
Observed
Ruminococcus
Prevotella
Bacteroides
clusterGroup.k3
Disease
non–T2DM
T2DM
B
***
*
**
***
ns
**
150
125
100
75
50
Observed
Ruminococcus
Prevotella
Bacteroides1
Bacteroides2
DMMcluster.k4
Disease
non–T2DM
T2DM

Figure S3

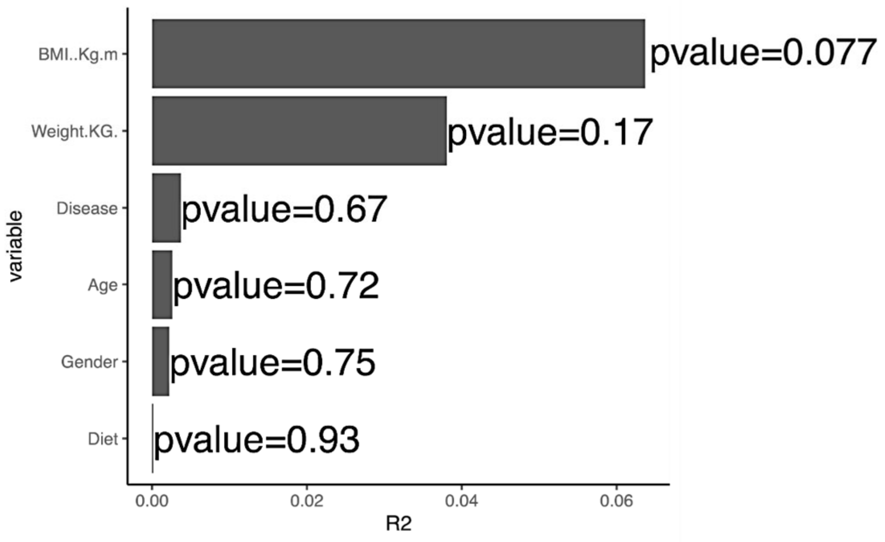
BMI..Kg.m
Weight.KG.
Disease
Age
Gender
Diet
variable
pvalue=0.077
pvalue=0.17
pvalue=0.67
pvalue=0.72
pvalue=0.75
pvalue=0.93
0.00
0.02
0.04
0.06
R2

Figure S4

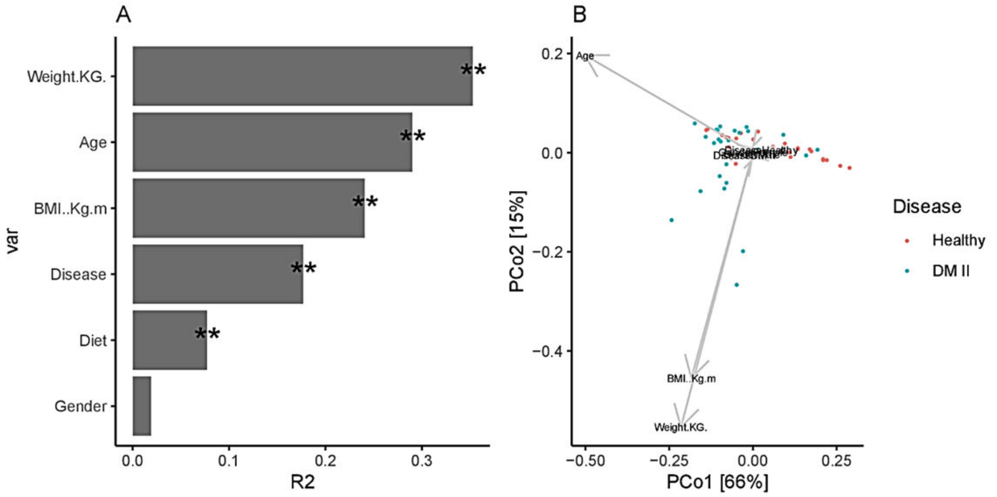
A
Weight.KG.
Age
BMI..Kg.m
Disease
Diet
Gender
var
R2
0.0
0.1
0.2
0.3
B
PCo2 [15%]
PCo1 [66%]
Age
BMI..Kg.m
Weight.KG.
−0.50
−0.25
0.00
0.25
0.2
0.0
−0.2
−0.4
Disease
Healthy
DM II

Figure S5
